# Impact of the Internal Carotid Artery Morphology on *in silico* Stent-Retriever Thrombectomy Outcome

**DOI:** 10.3389/fmedt.2021.719909

**Published:** 2021-08-03

**Authors:** Sara Bridio, Giulia Luraghi, Jose F. Rodriguez Matas, Gabriele Dubini, Giorgia G. Giassi, Greta Maggio, Julia N. Kawamoto, Kevin M. Moerman, Patrick McGarry, Praneeta R. Konduri, Nerea Arrarte Terreros, Henk A. Marquering, Ed van Bavel, Charles B. L. M. Majoie, Francesco Migliavacca

**Affiliations:** ^1^Laboratory of Biological Structure Mechanics, Department of Chemistry, Materials and Chemical Engineering, Politecnico di Milano, Milan, Italy; ^2^School of Engineering, National University of Ireland Galway, Galway, Ireland; ^3^Department of Biomedical Engineering and Physics, Amsterdam University Medical Center, Location Academic Medical Center, Amsterdam, Netherlands; ^4^Department of Radiology and Nuclear Medicine, Amsterdam University Medical Center, Amsterdam, Netherlands

**Keywords:** insist, finite element analysis, carotid siphon, acute ischemic stroke, internal carotid artery, digital twin

## Abstract

The aim of this work is to propose a methodology for identifying relationships between morphological features of the cerebral vasculature and the outcome of *in silico* simulations of thrombectomy, the mechanical treatment for acute ischemic stroke. Fourteen patient-specific cerebral vasculature segmentations were collected and used for geometric characterization of the intracranial arteries mostly affected by large vessel occlusions, i.e., internal carotid artery (ICA), middle cerebral artery (MCA) and anterior cerebral artery (ACA). First, a set of *global parameters* was created, including the geometrical information commonly provided in the clinical context, namely the total length, the average diameter and the tortuosity (length over head-tail distance) of the intracranial ICA. Then, a more exhaustive geometrical analysis was performed to collect a set of *local parameters*. A total of 27 parameters was measured from each patient-specific vascular configuration. Fourteen virtual thrombectomy simulations were performed with a blood clot with the same length and composition placed in the middle of the MCA. The model of TREVO ProVue stent-retriever was used for all the simulations. Results from simulations produced five unsuccessful outcomes, i.e., the clot was not removed from the vessels. The geometric parameters of the successful and unsuccessful simulations were compared to find relations between the vascular geometry and the outcome. None of the global parameters alone or combined proved able to discriminate between positive and negative outcome, while a combination of local parameters allowed to correctly identify the successful from the unsuccessful simulations. Although these results are limited by the number of patients considered, this study indicates a promising methodology to relate patient-specific geometry to virtual thrombectomy outcome, which might eventually guide decision making in the treatment of acute ischemic stroke.

## Introduction

The intra-arterial thrombectomy is a minimally invasive procedure based on stent-retriever technology for acute ischemic stroke (AIS) patients. An AIS arises when a large vessel occlusion caused by a thromboembolus (clot) prevents blood supply to the brain tissues. Despite the promising and encouraging results, complications may occur, such as thrombus fragmentation, vascular damage, or microembolization (1, 2).

**Figure 1:**
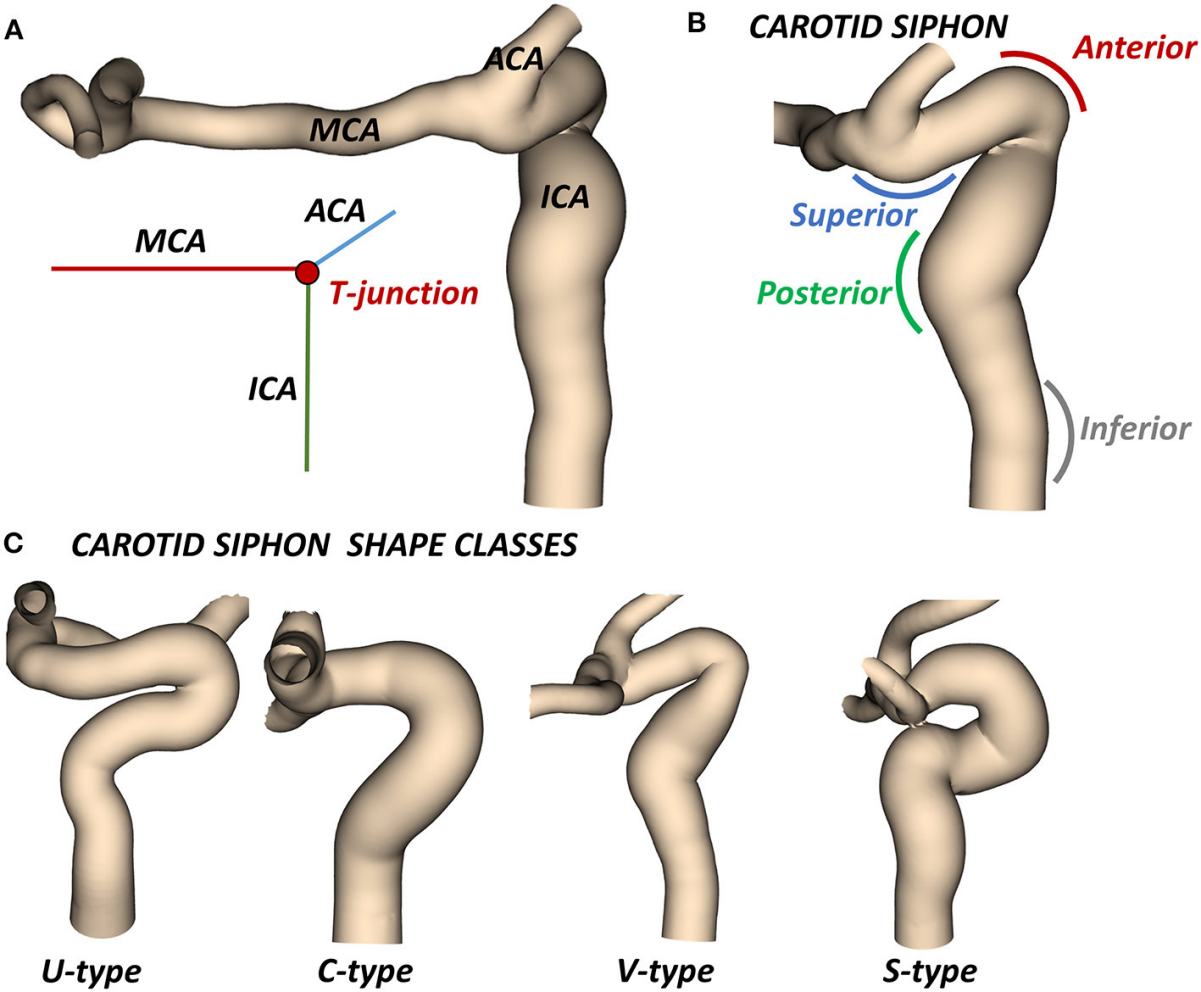
**(A)** Cerebral arteries mostly affected by AIS: the intracranial internal carotid artery (ICA) which bifurcates at the T-junction into the middle cerebral artery (MCA) and the anterior cerebral artery (ACA). **(B)** The carotid siphon with the superior, anterior, posterior, and inferior bends. **(C)** Classes of siphon shape.

The most likely location of the AIS is the intracranial internal carotid artery (ICA) and its bifurcated downstream segments. The main individual variability in ICA geometry can be found in the so-called carotid siphon (3). The carotid siphon lies between the carotid canal and the T-junction, where the ICA bifurcates into the middle cerebral artery (MCA) and the anterior cerebral artery (ACA) (Figure 1A). Four different bends can be recognized in the ICA (4, 5): (from the carotid canal) inferior, posterior, anterior, and superior (Figure 1B).

Although the way the ICA geometry affects the hemodynamics and different pathologies onset (aneurysms and stenosis) has been already investigated (6–10), the correlation between the ICA geometry and thrombectomy procedure outcome remains under-studied. Mokin et al. analyzed the correlation between the ICA geometric parameters and the outcome of the thrombectomy procedure, finding that the intracranial tortuosity did not influence the needed number of passes with thrombectomy devices (11). Srivatsa et al. investigated the influence of vessel morphology on the fist-pass success of mechanical thrombectomy in patients with MCA occlusions (12). Despite the limitation of performing manual measurements on 2D angiograms, they were able to conclude that a larger MCA diameter associated with a lower rate of tapering along the ICA was associated with high probability of first-pass recanalization. Schwaiger et al. observed an influence of MCA curvature on the success of recanalization performed with stent-retrievers (13). The curvature was expressed as angle between the ICA and the proximal part of the MCA and angle between proximal and distal MCA segments. Results showed that patients with unsuccessful recanalization had significantly higher angles. However, this study did not differentiate the patients based on other features like the thrombus length and composition or the type of stent-retriever used in the procedure, hence the causality between MCA curvature and successful recanalization could not be clearly assessed. Moreover, the tortuous geometry of the ICA was not considered in the study.

In the clinical application, the ICA is usually characterized by its average diameter, length, tortuosity (artery length over head-tail distance), or by other mean values (14). A classification based on the siphon shape is also used to evaluate the artery accessibility (15). In particular, the combined shape of the anterior and posterior bends defines four classes of siphon shape (U, C, V and S) (Figure 1C). Differently, two studies proposed an objective and extensive geometric characterization of carotid siphons, parametrized by a set of local geometric parameters (4, 7). Zhang et al. proposed to characterize the carotid siphon by identifying the two main bends and measuring their curvature, the lumen diameters at the end-points of each bend and the angle and distance between them. Bogunović et al. further enriched the carotid siphon analysis by dividing the ICA in four bends and measuring for each of them the curvature, the vessel diameter and the angles between adjacent bends.

From a technological perspective, the regulatory process for biomedical devices recently started receiving and accepting *in silico* evidence from modeling and numerical simulations (16). Recently our group developed and validated a finite-element model of the stent-retriever thrombectomy (17) for *in silico* clinical trials in AIS (18); this methodology was subsequently applied to make a replica of the procedure in a patient-specific case (19).

In this work, we hypothesize that local geometric features of the ICA are related to the virtual stent-retriever thrombectomy outcome. In particular, (i) a geometric characterization of 14 patient-specific cerebral vasculatures is carried out with 4 global and 27 local parameters for each patient; (ii) the thrombectomy procedure is simulated for each patient with the same settings (thrombus characteristics and stent-retriever); (iii) a preliminary post-processing correlation between the global and local parameters and the virtual thrombectomy outcome is proposed as methodology for future investigations.

## Methods

### Geometric Characterization

**Figure 2:**
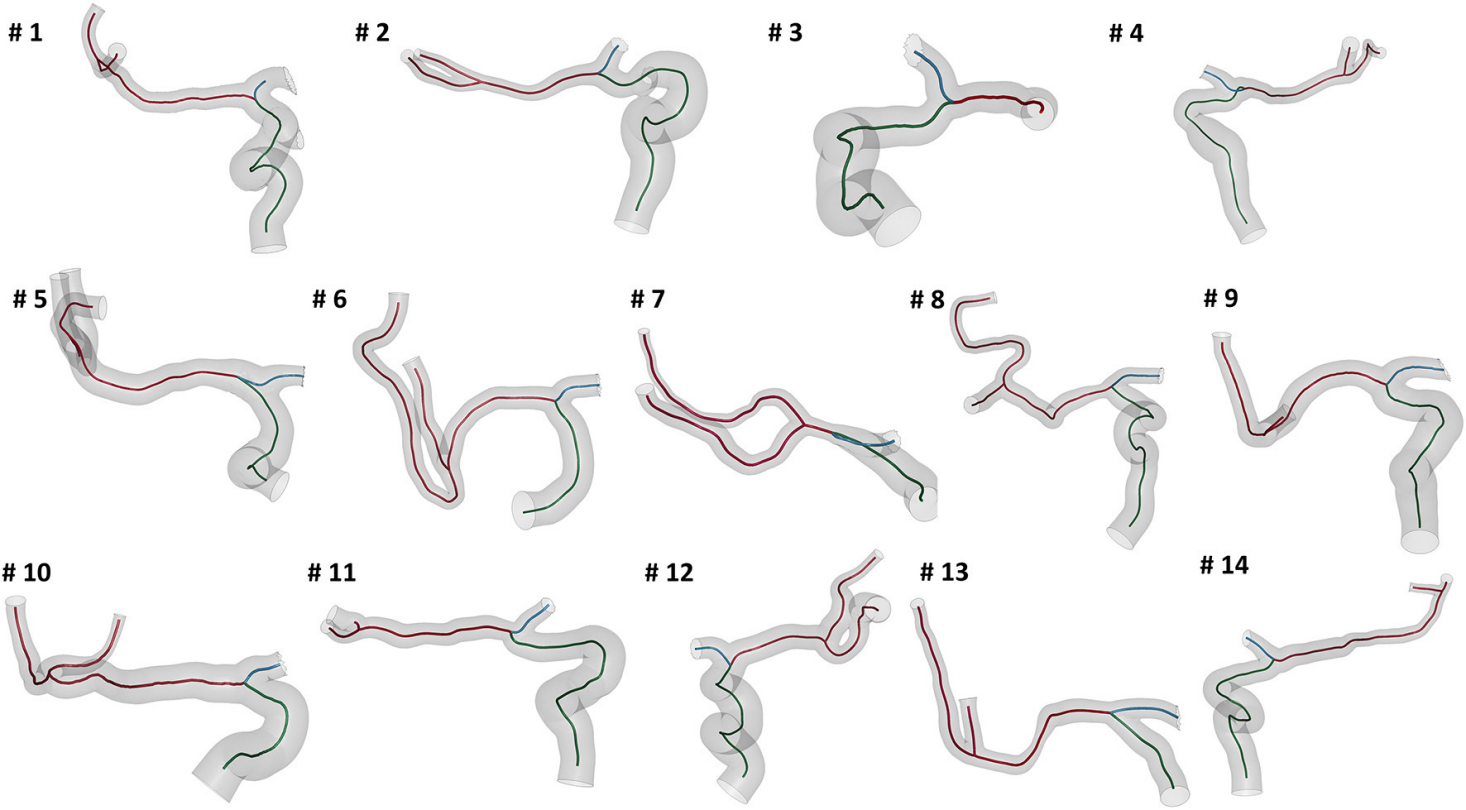
The reconstructed vessel surfaces and centerlines of the 14 studied patients: the vascular segments of interest are ICA (green), MCA (red) and ACA (blue).

We selected patients from the MR CLEAN (Multicenter Randomized Clinical Trial of Endovascular Treatment for Acute Ischemic Stroke in the Netherlands) Registry, which is an ongoing, prospective, observational, multicenter study, including data of stroke patients treated in 16 intervention hospitals in the Netherlands (20). The study population consisted of 14 patients who presented with an AIS due to a large vessel occlusion in the anterior circulation. The patients underwent a non-contrast computed tomography (NCCT) and a computed tomography angiography (CTA) scan before treatment. The segmentations of intracranial vessels were performed with StrokeViewer (NICO.LAB, Amsterdam, The Netherlands). iCAFE (14) (© 2016-2018 University of Washington. Used with permission.), a semi-automated software, was used to extract centerlines, local radius, and to label arterial segments. For a more detailed description of the vascular segmentation and centerline computation, refer to (21). The centerlines of the arteries of interest, i.e. ICA, MCA, and ACA, were extracted (Figure 2) and analyzed to obtain the values of the geometric features describing each patient-specific vessel geometry.

First, the geometric parameters routinely considered in the clinical context were measured for each patient geometry. These parameters are the total length of the intracranial ICA just distal form the carotid canal to the T-junction (*L*^*ICA*^), the average diameter of the ICA (*D*^*ICA*^) and the ICA tortuosity (*tor*^*ICA*^), calculated as (22):


$$\begin{array}{l} tor^{ICA}=\frac{L^{ICA}}{d^{ICA}}-1 \end{array}$$

where *d*^*ICA*^ is the Euclidean distance of the ICA endpoints. Additionally, each ICA siphon was visually classified into U, C, V, or S shapes. In this work, this set of parameters is labeled as *Global parameters* since they are referred to the entire ICA geometry.

**Figure 3:**
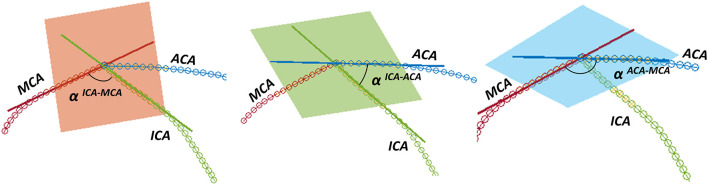
Measurement of the angles at the T-junction: from left to right, α^ICA−MCA^, α^ICA−ACA^, α^ACA−MCA^.

**Figure 4:**
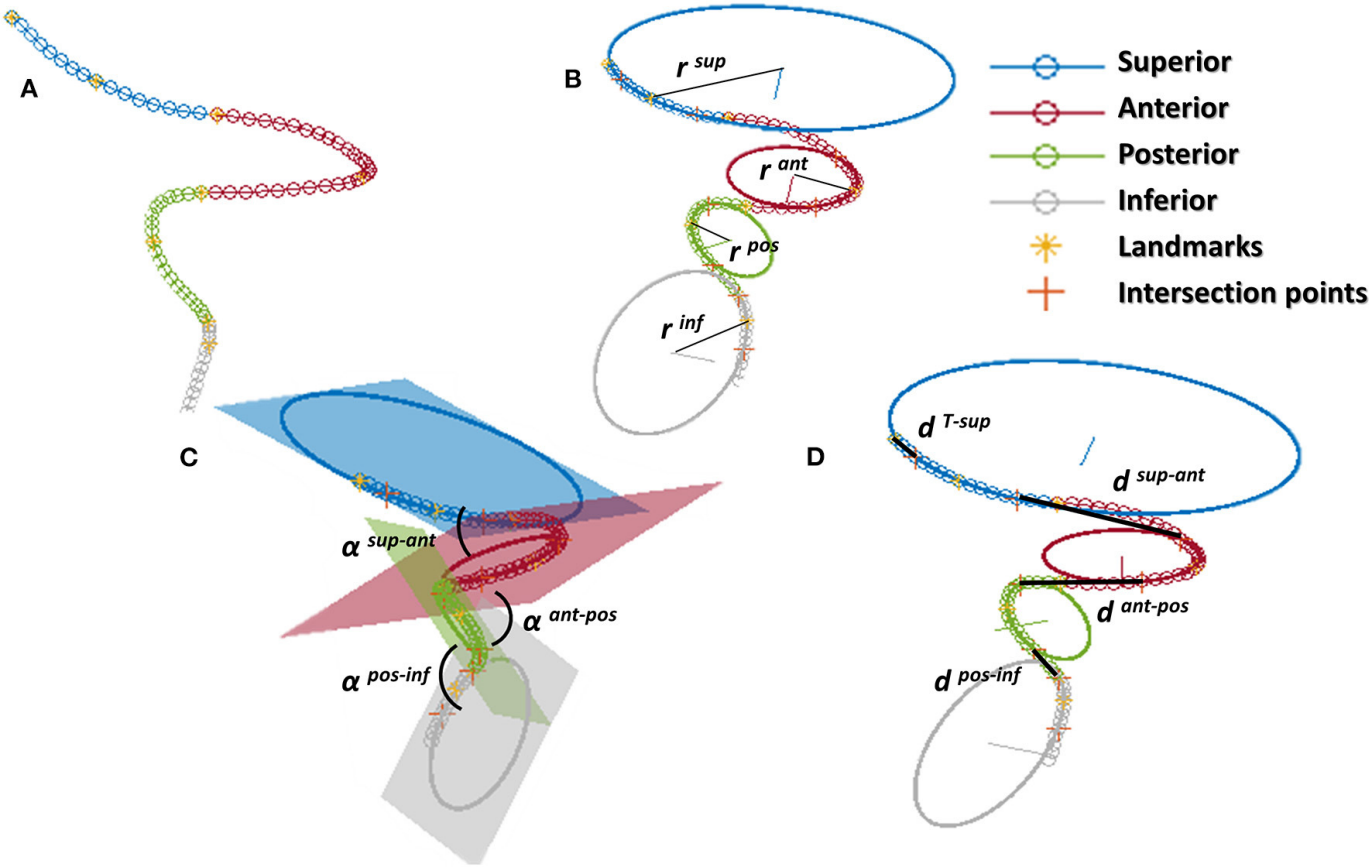
ICA analysis: **(A)** division of the ICA in the 4 bends (superior, anterior, posterior, inferior) separated by the landmarks; **(B)** calculation of curvature of each bend as the radius of the fitting circle; **(C)** angles between bends; **(D)** distance between bends calculated at adjacent intersection points between the bend and the relative fitting circle; for the superior bend, the distance is calculated between the distal intersection point and the ICA endpoint.

Successively, the patient-specific vessel geometries were analyzed to extract the set of *Local parameters*. This process was made fully automatic by means of a MATLAB (R2020a, The MathWorks, Natick, MA, USA) script. First, the angles created at the T-junction were measured (*α*^*ICA*−*MCA*^, *α*^*ICA*−*ACA*^, *α*^*ACA*−*MCA*^). Each angle was measured by fitting the two segments of the vessels converging into the T-junction with a linear segment and by measuring the angle formed in the plane containing both the linear segments (Figure 3). The ICA shape was then analyzed. Following the method proposed in (4), each ICA centerline was divided into four segments: superior, anterior, posterior, and inferior bends, progressively located from the T-junction to the end closest to the heart (Figure 4A). The landmarks dividing adjacent bends were identified with a combined use of the Frenet-Serret triad and a parallel transport frame following the ICA curve (4). The four bends and couples of adjacent bends were analyzed to obtain the following geometric parameters fully describing the ICA geometry:

- bends lengths: *L*^*sup*^, *L*^*ant*^, *L*^*pos*^, *L*^*inf*^;
- bends diameters: *D*^*sup*^, *D*^*ant*^, *D*^*pos*^, *D*^*inf*^, measured at the point of maximum bend curvature;
- curvature radii: *r*^*sup*^, *r*^*ant*^, *r*^*pos*^, *r*^*inf*^, calculated as the radius of the circle best fitting the bend (Figure 4B);
- bends tortuosity: *tor*^*sup*^, *tor*^*ant*^, *tor*^*pos*^, *tor*^*inf*^, calculated with the formula:
  $$\begin{array}{l} tor^{bend}=\frac{L^{bend}}{d^{bend}}-1 \end{array}$$
- where *L*^*bend*^ is the length of the bend and *d*^*bend*^ is the Euclidean distance of the bend endpoints;
- angles between adjacent bends: *α* ^*sup*−*ant*^, *α* ^*ant*−*pos*^, *α* ^*pos*−*inf*^, measured between the planes containing the fitting circle of each bend (Figure 4C);
- distance between the bifurcation point and the starting point of the superior bend (considered as the more distal intersection point between the superior bend and its fitting circle): *d*^*T*−*sup*^ (Figure 4D);
- distances between adjacent bends: *d*^*sup*−*ant*^, *d*^*ant*−*pos*^, *d*^*pos*−*inf*^, calculated as the distance between the ending point of the more distal bend and the starting point of the more proximal one, where the starting and ending points of each bend are considered as the distal and proximal intersection point of the bend and the relative fitting circle, respectively (Figure 4D).

Finally, the average diameter of the MCA (*D*^*MCA*^) was calculated. The *Local parameters* set thus consists of 27 parameters for each patient-specific vessel geometry.

### Virtual Thrombectomy Procedure

To study the impact of the geometric features of the intracranial ICA on the virtual thrombectomy procedures, the same thrombus, stent-retriever, and thrombectomy procedure were implemented in the 14 patient-specific vessel geometries.

The patient-specific domains were obtained from the computed centerlines with the aid of level-set images (21). In particular, triangular shell elements with a mean element size of 0.2 mm were used to discretized the vessel walls using the open-source MATLAB toolbox GIBBON (23). Rigid wall material was assumed.

**Figure 5:**
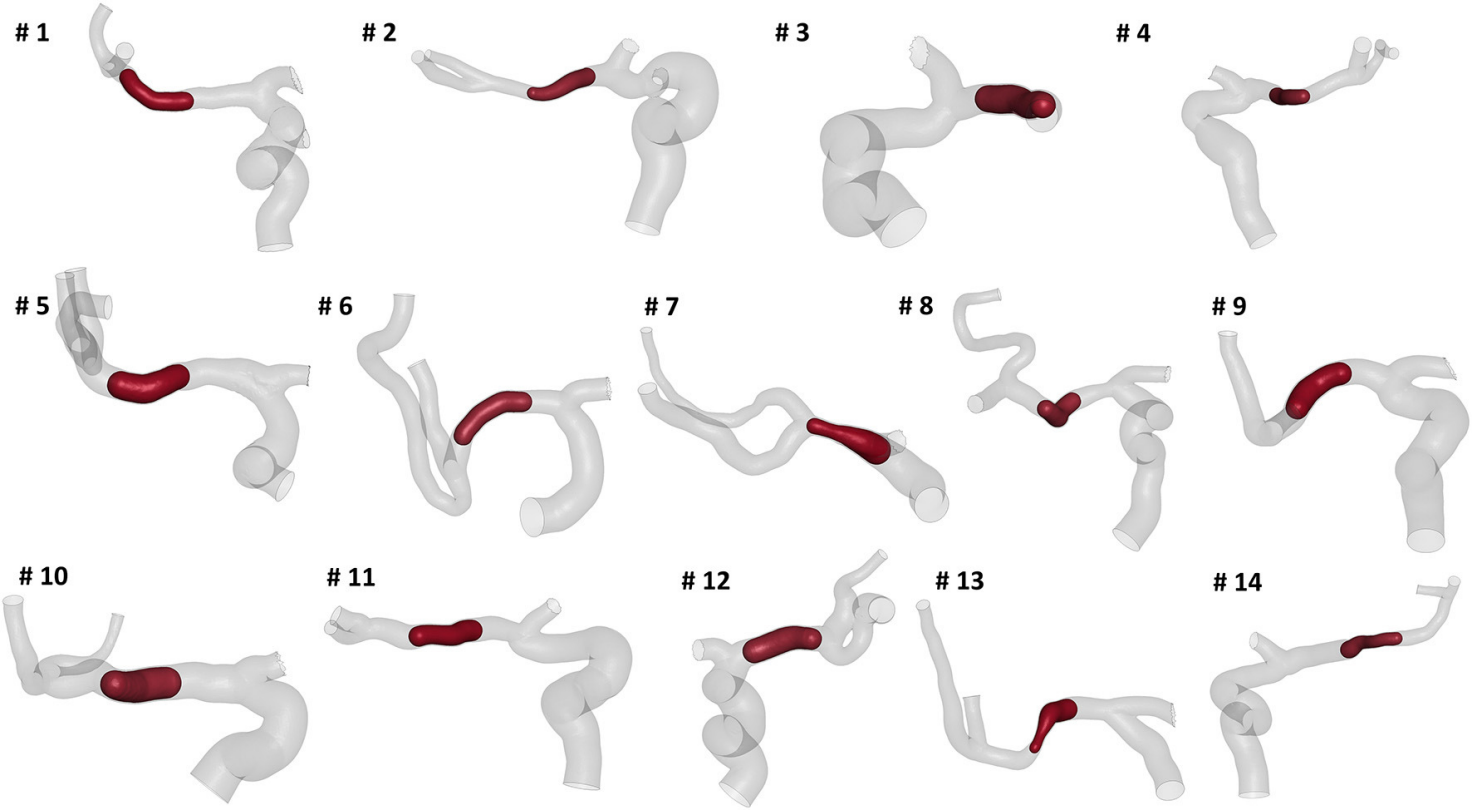
A clot of 14mm of length placed in the central MCA segment of the 14 patients.

Thrombi with the same length and composition (in terms of red blood cells (RBC) and fibrin contents) were placed in the central part of the MCA (before its bifurcation) for each patient (Figure 5), as this is the most common AIS location (24). The thrombus characteristics were chosen as mean values obtained from the literature. In particular with a length of 14 mm (25) and 35% RBC - 65% fibrin composition (26). The thrombus diameter was set to occlude 90% of the patient-specific MCA diameter where it is located. Thrombi were discretized using ANSA Pre Processor (BETA CAE System, Switzerland) with linear tetrahedral elements with a mean element size of 0.2 mm on the surface. The thrombus non-linear material was modeled with a quasi-hyperelastic foam formulation by fitting stress-strain curves from *in vitro* tests on *ex vivo* clots (19).

**Figure 6:**
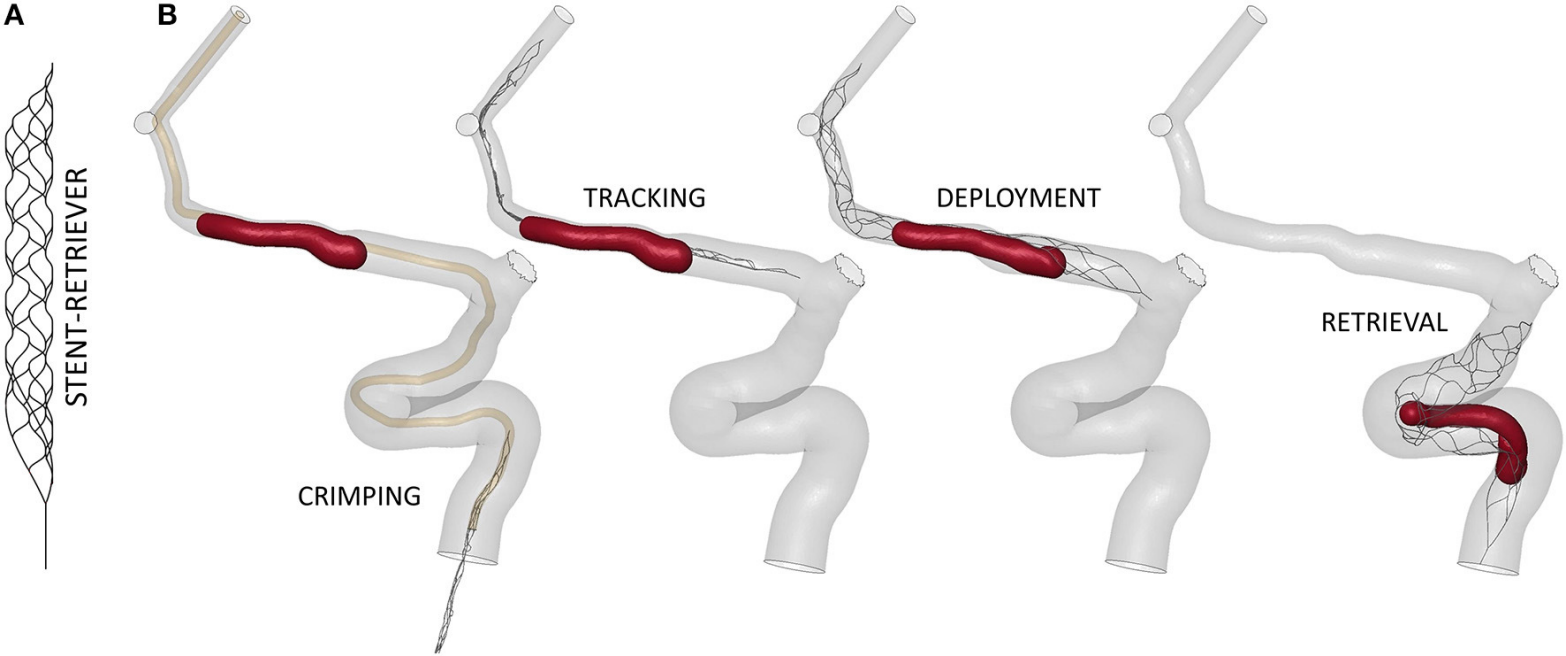
**(A)** Finite-element model of the TREVO ProVue 4–20mm stent-retriever. **(B)** The main steps of the thrombectomy simulation.

The geometry of the TREVO ProVue 4–20mm stent (Stryker, US) was obtained using a Python code and discretized with linear beam elements with a mean element length of 0.2 mm (Figure 6A). Hughes-Liu formulation with cross-section integration was used. A shape memory alloy material model was used to simulate the NiTi superelastic behavior. Details about the NiTi material parameter definitions can be found in (17).

The virtual thrombectomy procedure was simulated in the 14 patients with the same settings (Figure 6B): the stent-retriever is crimped in a 0.5mm diameter catheter and tracked to the clot location where it is deployed; finally, it is retrieved to extract the trapped clot. For a more detailed description of the contact algorithms, time step, damping system, and other technical details refer to (17), where a validation of the thrombectomy simulation with *in vitro* experiments was carried out. In Luraghi et al. (19) the same simulation settings were used to successfully reproduce a clinical case. The simulations were performed on a system featuring 40 CPUs (Intel Xeon64) and 256 GB of RAM, using the commercial finite-element solver LS-DYNA (ANSYS, USA) and lasted about 24 h.

### Post-processing Analysis

The virtual thrombectomy procedure was defined to have a positive outcome if the clot reaches the end of the ICA segment with the stent, and a negative outcome if the clot remains inside the vessel. The outcomes of the 14 simulations were investigated and correlated with the corresponding ICA geometric parameters and their combinations, with both the global and local sets of parameters.

## Results

### ICA Geometric Features

**Figure 7:**
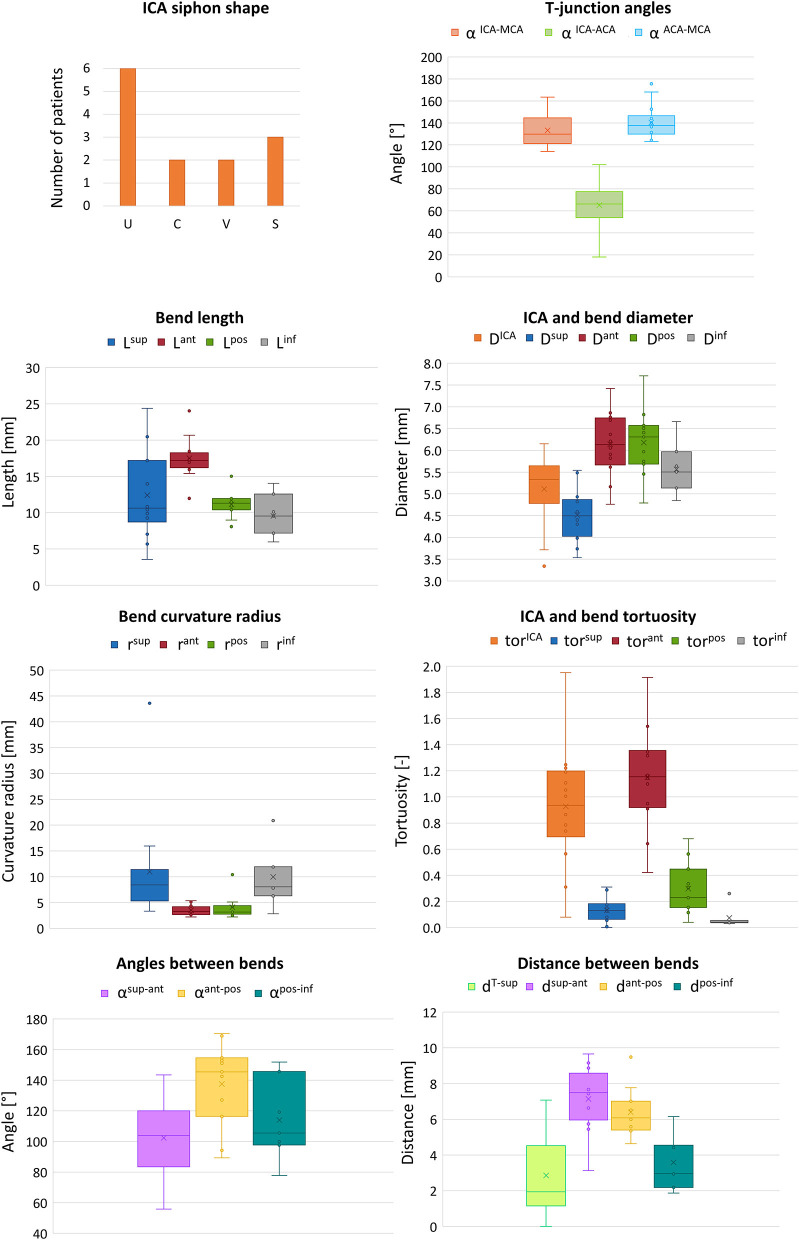
Box plots representing the distribution of the local and global geometric parameters.

The measured mean and local sets of geometric parameters are listed in Table 1. A few patients missed some of the bends to perform the local geometric analysis because the most proximal part of the ICA was not included or not discernible in the CTA images: the models of patients 3, 5, 9, and 10 do not include the inferior bend, patient 7 does not have the posterior and inferior bends, patients 6 and 13 do not have the anterior, posterior and inferior bends. In Figure 7, the box plots of the local and global geometric parameters are shown.

**Table 1:** Global and Local parameters of the 14 patient-specific geometries.

**Patient**	**# 1**	**# 2**	**# 3**	**# 4**	**# 5**	**# 6**	**# 7**	**# 8**	**# 9**	**# 10**	**# 11**	**# 12**	**# 13**	**# 14**
**Global parameters**														
*L^*ICA*^* (mm)	46.0	51.2	48.7	49.2	34.9	24.4	23.4	52.9	48.3	30.2	50.3	45.3	17.2	51.1
*D^*ICA*^* (mm)	5.9	5.5	5.2	5.5	5.6	4.9	3.7	4.8	6.1	5.6	4.9	5.7	3.3	4.7
*tor^*ICA*^* (-)	0.9	1.1	1.9	0.7	0.8	0.3	0.6	1.0	1.0	0.9	1.2	1.2	0.1	1.2
*Siphon*	S	S	U	V	V	C	C	U	U	U	U	S	–	U
**Local parameters**														
*α^*ICA*−*MCA*^* (°)	123	153	119	115	142	124	158	122	163	136	136	128	132	114
*α^*ICA*−*ACA*^* (°)	76	68	102	60	40	90	18	57	64	79	63	77	44	72
*α^*ACA*−*MCA*^* (°)	131	136	124	152	168	144	176	131	132	144	140	123	139	125
*L^*sup*^* (mm)	9.5	9.9	20.5	7.0	14.0	24.4	5.7	14.2	9.3	3.5	10.8	10.4	17.2	17.3
*L^*ant*^* (mm)	17.3	20.7	16.9	17.2	12.0	–	17.7	17.2	24.0	15.4	18.5	17.1	–	15.9
*L^*pos*^* (mm)	11.9	8.1	11.3	10.9	9.0	–	–	11.3	15.0	11.3	11.5	10.4	–	11.9
*L^*inf*^* (mm)	7.2	12.6	–	14.0	–	–	–	10.1	–	–	9.5	7.4	–	6.0
*D^*sup*^* (mm)	4.9	4.4	4.6	3.7	4.8	4.8	3.5	4.3	5.5	4.6	4.3	5.5	4.0	4.0
*D^*ant*^* (mm)	7.4	6.4	6.1	4.8	6.9	–	5.2	5.6	6.7	5.8	6.2	6.8	–	5.9
*D^*pos*^* (mm)	6.3	6.6	5.7	7.7	6.0	–	–	4.8	6.8	5.7	6.4	6.5	–	5.5
*D^*inf*^* (mm)	5.6	6.7	–	5.5	–	–	–	5.2	–	–	5.1	6.0	–	4.8
*r^*sup*^* (mm)	8.7	4.0	10.6	3.3	8.1	8.5	13.7	5.1	5.4	43.6	8.3	7.9	10.7	16.0
*r^*ant*^* (mm)	3.3	4.4	3.7	2.6	2.2	–	5.4	2.7	5.1	3.6	2.7	3.3	–	3.2
*r^*pos*^* (mm)	2.4	2.2	4.4	5.1	4.3	–	–	4.3	10.4	3.2	2.5	3.0	–	2.8
*r^*inf*^* (mm)	6.3	8.1	–	11.9	–	–	–	7.8	–	–	12.0	2.9	–	20.9
*tor^*sup*^* (-)	0.08	0.15	0.19	0.29	0.13	0.31	0.01	0.18	0.17	0.00	0.14	0.06	0.08	0.07
*tor^*ant*^* (-)	1.14	1.92	0.95	1.32	0.64	–	0.42	1.54	1.10	0.91	1.36	1.17	–	1.34
*tor^*pos*^* (-)	0.68	0.34	0.17	0.15	0.11	–	–	0.23	0.04	0.31	0.23	0.45	–	0.56
*tor^*inf*^* (-)	0.04	0.05	–	0.04	–	–	–	0.04	–	–	0.05	0.26	–	0.03
*α^*sup*−*ant*^* (°)	74	97	91	115	80	–	143	105	102	134	106	121	–	56
*α^*ant*−*pos*^* (°)	94	127	153	170	90	–	–	155	145	169	116	142	–	152
*α^*pos*−*inf*^* (°)	146	100	–	98	–	–	–	105	–	–	119	78	–	152
*d^*T*−*sup*^* (mm)	1.8	3.6	5.7	1.3	2.1	6.7	0.6	4.1	0.2	0.0	1.6	1.5	3.64	7.1
*d^*sup*−*ant*^* (mm)	7.7	7.4	6.6	7.7	7.5	–	3.1	9.2	8.9	5.4	9.7	6.7	–	5.7
*d^*ant*−*pos*^* (mm)	5.6	5.3	7.0	7.8	6.1	–	–	6.5	4.6	6.0	9.5	6.6	–	5.4
*d^*pos*−*inf*^* (mm)	2.9	4.4	–	6.2	–	–	–	3.0	–	–	1.9	2.2	–	4.5
*D^*MCA*^* (mm)	3.5	2.8	3.2	3.4	5.3	3.5	2.1	3.7	4.4	4.2	3.1	4.4	2.2	2.4

### Virtual Thrombectomy Outcome

**Figure 8:**
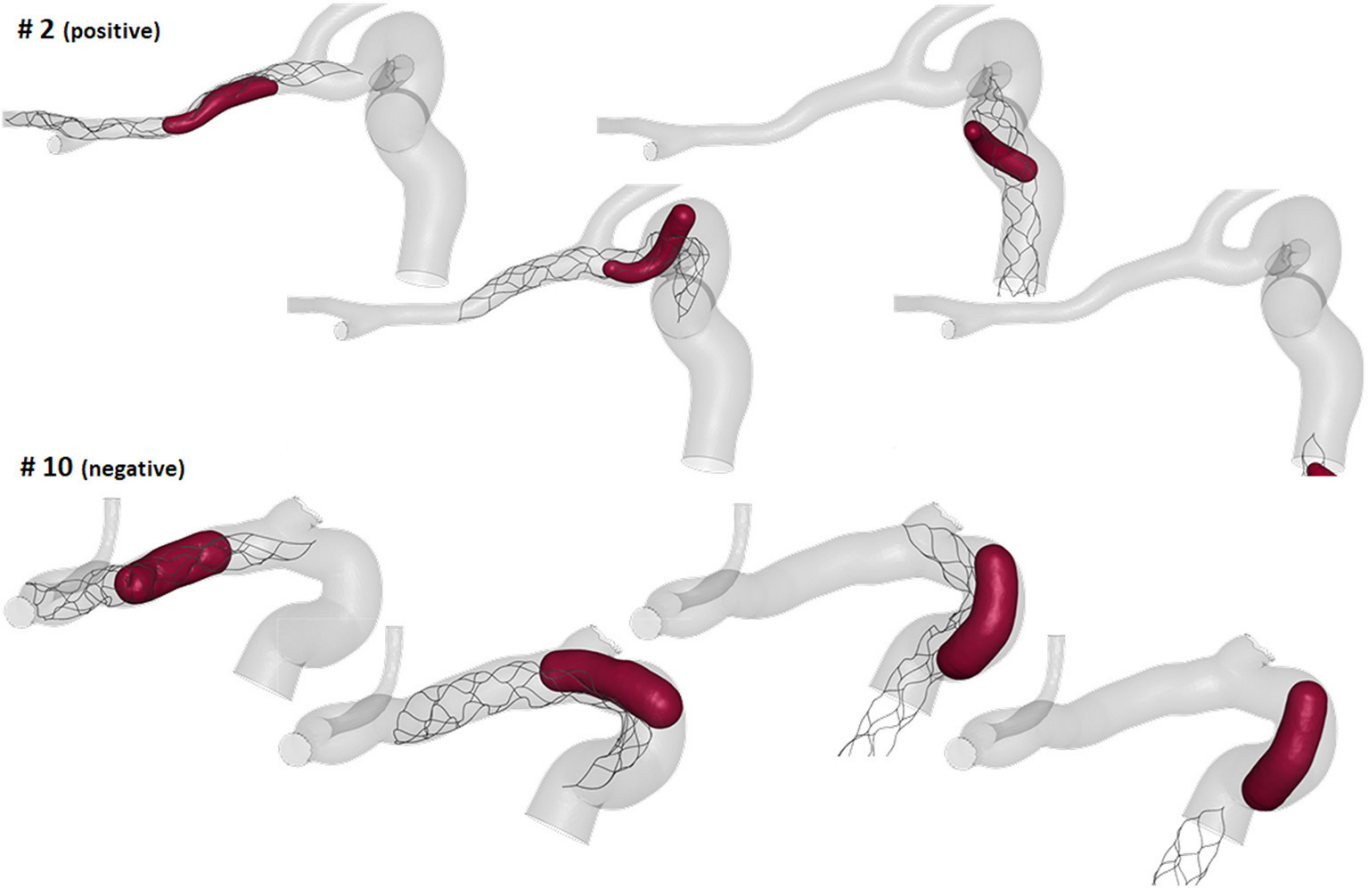
Examples of virtual thrombectomies with positive (**top**) and negative (**bottom**) outcome.

The thrombectomy simulations presented with following results: nine had a positive outcome and five a negative outcome. In the successful thrombectomy simulations (patients 1, 2, 3, 6, 7, 9, 11, 13, and 14) the clots remained captured in the stent-retriever during the retrieval phase and reached the end of the ICA segment (Figure 8-top). On the contrary, in the failed thrombectomies (patients 4, 5, 8, 10, and 12), the clots lost contact with the stent-retriever and remained in the anterior bend of the ICA (Figure 8-bottom).

### Correlation Between ICA Geometry and Thrombectomy Outcome

The outcomes of the thrombectomy simulations were analyzed with the associated global and local geometric parameters to find indicators able to determine the positive or negative outcome. Associations with the thrombectomy outcomes were analyzed for each parameter singularly and for combinations of parameters within each group (global or local). Only the significant associations are here presented.

**Figure 9:**
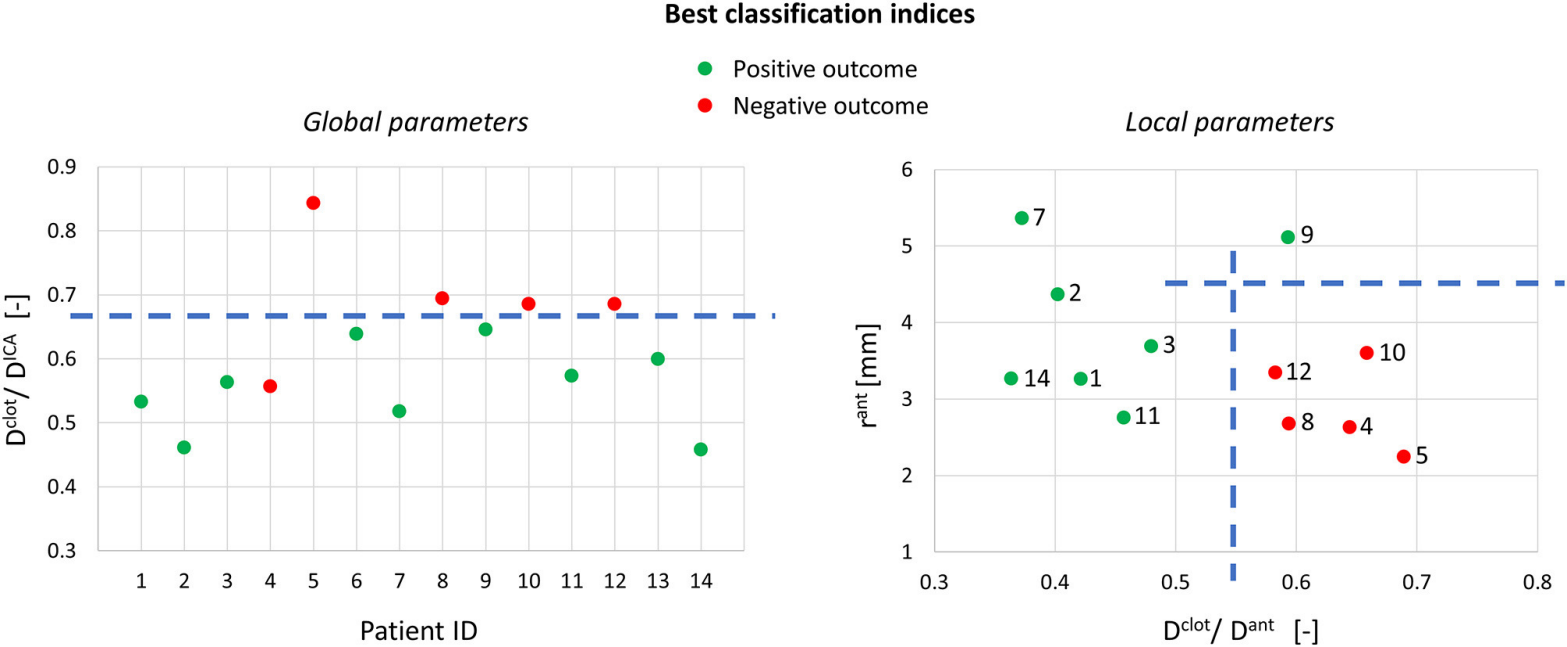
Best classification indices using the global and local geometric parameters (on the right, only 12 samples are shown because the segmentation for patients 6 and 13 did not include the anterior bend).

Using the global geometric parameters, the index with the best performance in discriminating positive and negative cases is the ratio between the clot diameter (90% of MCA diameter) and the average ICA diameter, *D*^*clot*^*/D*^*ICA*^ (Index 1 in Table 2), with a threshold of 0.67 (calculated as the mean value between the two patients closest to the boundary, i.e., patients 9 and 12) able to correctly classify 13 cases out of 14 (Figure 9-left). However, besides the misclassified case (patient 4), many correctly classified samples are very close to the separating boundary, indicating that the threshold is not robust in discriminating the cases.

**Table 2:** Indices used for correlating the thrombectomy simulation outcome with global and local geometric parameters of the patient (+ positive outcome, –negative outcome).

**Patient**	**# 1**	**# 2**	**# 3**	**# 4**	**# 5**	**# 6**	**# 7**	**# 8**	**# 9**	**# 10**	**# 11**	**# 12**	**# 13**	**# 14**
Outcome	+	+	+	–	–	+	+	–	+	–	+	–	+	+
D^clot^ (mm)	3.1	2.6	2.9	3.1	4.7	3.1	1.9	3.3	4.0	3.8	2.8	3.9	2.0	2.1
Index1 D^clot^/D^ICA^	0.53	0.46	0.56	0.56	0.84	0.64	0.52	0.69	0.65	0.69	0.57	0.68	0.60	0.46
Index2 D^clot^/D^ant^	0.42	0.40	0.48	0.64	0.69	–	0.37	0.59	0.59	0.66	0.46	0.58	–	0.36
r^ant^ (mm)	3.3	4.4	3.7	2.6	2.2	–	5.4	2.7	5.1	3.6	2.7	3.3	–	3.2

Considering the local geometric parameters, a different index looked more promising to our aim. The combination of the ratio between the clot diameter and the diameter of the anterior bend in the point of maximum curvature, *D*^*clot*^*/D*^*ant*^ (Index 2 in Table 2), and the curvature radius of the anterior bend, *r*^*ant*^, allowed for the identification of a separation boundary which correctly classifies the cases, with a wide margin between the two classes (Figure 9-right). For the patients considered in this study, thresholds around 0.55 for *D*^*clot*^*/D*^*ant*^ and 4.5 mm for *r*^*ant*^ were identified. Namely, when the clot diameter is large with respect to the diameter of the anterior bend in the point of its maximum curvature (*D*^*clot*^*/D*^*ant*^ > 0.55) and the anterior bend has a pronounced curvature (*r*^*ant*^ < 4.5mm), the stent is not able to remove the clot from the anterior bend, which is the failure mode observed in all the 5 cases with a negative outcome.

## Discussion

The proposed geometrical analysis of the ICA is aimed at suggesting an association between geometrical features and *in silico* thrombectomy outcome.

**Figure 10:**
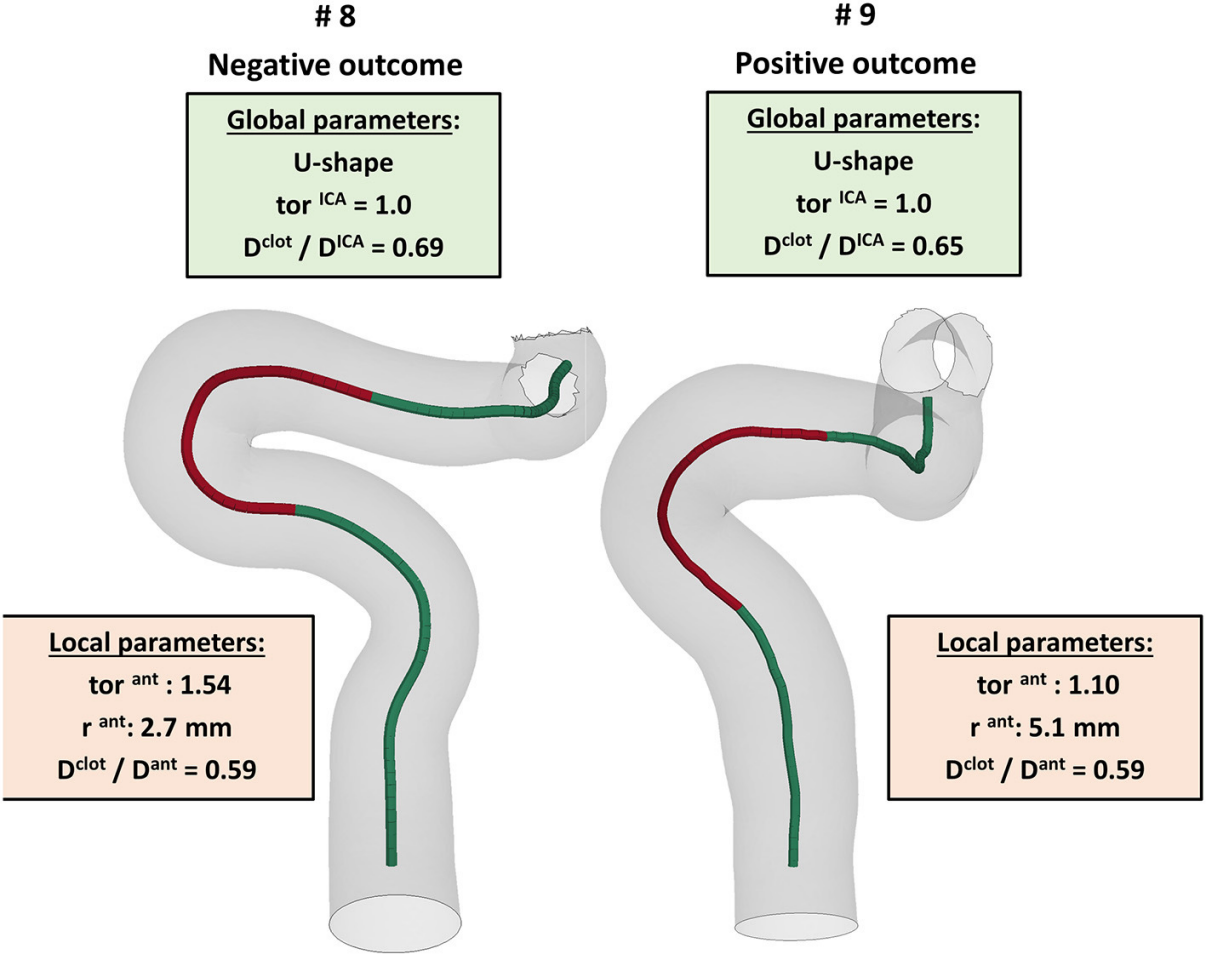
Example of patients, 8 and 9, presenting very similar global parameters (green boxes), but different local parameters (red boxes), with a particular focus on the anterior bend (red tract of the ICA centerline), which resulted in different virtual thrombectomy outcomes.

The first aspect this study has highlighted is the importance of analyzing the local parameters rather than the global ones to discriminate the different morphologies of the ICA. For example, while the global parameters (siphon shape and ICA tortuosity) for patients 8 and 9 are the same, the morphology of the ICA bends is clearly different, as shown in Figure 10. Focusing on the anterior bend, the curvature radius appears to be a better index of the bend morphology with respect to the tortuosity index. Again, considering patients 8 and 9 as examples, the difference in the curvature radius (2.7 vs. 5.1 mm) is much more important than the difference in the tortuosity index (1.54 vs. 1.10). It is to be noted that patients 8 and 9 present a different virtual thrombectomy outcome.

As regards the *in silico* thrombectomies, the set of local geometric parameters proved to be more efficient in identifying indices able to discriminate positive and negative simulation outcomes with respect to the global parameters. In particular, the anterior bend was identified as the segment of the ICA determining the possible failure of the procedure. A combination of a high ratio between the diameters of the blood clot and of the anterior bend and a small curvature radius of the anterior bend appears to be a decisive feature for negative *in silico* thrombectomy outcomes. Comparing again patients 8 and 9, from Table 2 it can be observed that since these cases have the same value for the index D^clot^/D^ant^, the outcome cannot be correctly distinguished from this index alone. However, when the radius of the anterior bend curvature in patient 8 is also considered, failure to remove the blood clot appears to be predictable (Figures 9, 10). On the other hand, patients 8 and 11 present with the same value of anterior bend curvature, however, here the difference in the outcome (Figure 9) appears to be due to the relatively large size of the clot with respect to the anterior bend diameter in patient 8. In this study, the indicated threshold values represent only an indication and they should be refined considering more patients with geometric features close to the separating boundaries. Furthermore, the findings presented may be limited to the particular type of thrombus and stent-retriever modeled. Indeed, this study was conducted using a single type of blood clot (65% fibrin content and 14 mm length) and one single stent-retriever design (TREVO ProVue).

A limitation of this work is the small number of patients: future work will gather more patient-specific cerebral vasculature geometries to refine this study. Nonetheless, the 14 patients had substantially different siphon shapes and geometric features, which therefore represented a broad spectrum of vascular geometries. Furthermore, the calculated global and local parameters shown in Figure 7 are in line with what was reported in the literature (4, 7, 24), indicating that the 14 patients of this study are a good representation of the AIS population. The lack of some bends in a subset of patients, due to truncated segmentations of the ICA, is another limitation. In addition, it has not been possible to compare the outcomes of the simulations with the actual outcomes of the clinical procedures. This is because in this study, to focus solely on the impact of the ICA geometry, all simulations were conducted using the same type of retriever device and the same clot position and composition. In reality, however, each patient may have clinically presented with a different type of clot in a different location and may have been treated with a different retriever device. Therefore, the correlations reported here can only be associated with *in silico* thrombectomy. However, the results presented do shed light on the potential role that the local geometric parameters of the ICA, together with the geometric and mechanical characteristics of the clot and the retriever, may play on predicting the outcome of thrombectomy in clinic. Additionally, this study focused on the ability of the considered stent-retriever to remove the thrombus from the cerebral vessels. However, the recanalization may also be incomplete due to thrombus fragmentation, which can cause embolization. The fragmentation of the clot was not modeled in the simulations. Finally, the vessel was modeled with a rigid material, hence vascular damage due to the procedure could not be assessed. The integration in the simulation of mechanisms of thrombus fragmentation, deformable vessel walls and the introduction of the blood flow to simulate procedures without the use of a balloon guide catheter will be integrated in the future to improve the impact of similar studies.

Future work should investigate the influence of the clot features and the effect of using a different stent-retriever or a different thrombectomy technique on the conducted analyses. Once this analysis is made, it might be used in the clinic in the pre-planning phase to select the best stent-retriever, catheters and procedure for a selected patient. Indeed, once the vessel segmentation process is performed, the implemented automatic script can provide in real-time the patient's set of local parameters needed to assess the most suitable thrombectomy procedure. In this process, the main user-time consuming step would be the image segmentation. The results of this work pave the way to find an association between the virtual thrombectomy outcome and the local set of parameters, rather than the global parameters. This study is not intended to be conclusive, but it can be considered as a first step toward a classification strategy to be used for decision making in the treatment of AIS patients.

## Data Availability Statement

The datasets presented in this article are not readily available because this dataset is property of the INSIST consortium. Requests to access the datasets should be directed to https://www.insist-h2020.eu/index.php/contact-us.

## Author Contributions

SB and GL designed the study and drafted the manuscript. JR and GD supported the implementation of the study and participated the analysis and the discussion of results. GG and GM carried out the thrombectomy simulations. JK implemented the automatic script to analyze the geometry. KM processed the segmentations and provided the discretized patient-specific geometries with the supervision of PM. PK and NA performed the segmentation process with the supervision of HM. HM, EB, and CM helped in supervising the research project and gave insights for the clinical aspects and revise the manuscript. FM was in charge of overall direction and planning. All authors contributed to the article and approved the submitted version.

## Conflict of Interest

HM reports co-founder and shareholder of Nico.lab, a company that focuses on the use of artificial intelligence for medical image analysis. CM received funds from the European Commission (related to this project, paid to institution); and from CVON/Dutch Heart Foundation, Stryker, TWIN Foundation, Health Evaluation Program Netherlands (unrelated; all paid to institution). CM is shareholder of Nico.lab, a company that focuses on the use of artificial intelligence for medical imaging analysis. The remaining authors declare that the research was conducted in the absence of any commercial or financial relationships that could be construed as a potential conflict of interest.

## Publisher's Note

All claims expressed in this article are solely those of the authors and do not necessarily represent those of their affiliated organizations, or those of the publisher, the editors and the reviewers. Any product that may be evaluated in this article, or claim that may be made by its manufacturer, is not guaranteed or endorsed by the publisher.

## Appendix

### INSIST Investigators

Charles Majoie^1^, Henk Marquering^1,2^, Ed van Bavel^2^, Alfons Hoekstra^3^, Diederik W.J. Dippel^4^, Hester L. Lingsma^5^, Aad van der Lugt^6^, Noor Samuels^4,5,6^, Nikki Boodt^4,5,6^, Yvo Roos^7^, Simon de Meyer^8^, Senna Staessens^8^, Praneeta Konduri^1,2^, Nerea Arrarte Terreros^2^,Bastien Chopard^9^, Franck Raynaud^9^, Remy Petkantchin^9^, Vanessa Blanc-Guillemaud^10^, Mikhail Panteleev^11,12^, Alexey Shibeko^11^, Francesco Migliavacca^13^, Gabriele Dubini^13^, Giulia Luraghi^13^, Jose Felix Rodriguez Matas^13^, Sara Bridio^13^, Patrick Mc Garry^14^, Kevin Moerman^14^, Behrooz Fereidoonnezhad^14^, Michael Gilvarry^15^, Ray McCarthy^15^, Sharon Duffy^15^, Stephen Payne^16^, Tamas Jozsa^16^, Wahbi El-Bouri^13^, Sissy Georgakopoulou^2^, Victor Azizi^3^, Raymond Padmos^3^, Anushree Dwivedi^15^

^1^ Department of Radiology and Nuclear Medicine, Amsterdam UMC, location AMC, Amsterdam, Netherlands

^2^ Biomedical Engineering and Physics, Amsterdam UMC, location AMC, Amsterdam, Netherlands

^3^ Computational Science Lab, Faculty of Science, Institute for Informatics, University of Amsterdam, Amsterdam, Netherlands

^4^ Department of Neurology, Erasmus MC University Medical Center, Rotterdam, Netherlands

^5^ Department of Public Health, Erasmus MC University Medical Center, Rotterdam, Netherlands

^6^ Department of Radiology & Nuclear Medicine, Erasmus MC University Medical Center, Rotterdam, Netherlands

^7^ Department of Neurology, Amsterdam UMC, location AMC, Amsterdam, Netherlands

^8^ Laboratory for Thrombosis Research, KU Leuven Campus Kulak Kortrijk, Kortrijk, Belgium

^9^ Computer Science Department, University of Geneva, CUI, Carouge, Switzerland

^10^ Institut de Recherches Internationales Servier, Coubevoie Cedex, France

^11^ Center for Theoretical Problems of Physicochemical Pharmacology RAS, Moscow, Russia

^12^ Dmitry Rogachev National Research Center of Pediatric Hematology, Oncology and Immunology, Moscow, Russia; Faculty of Physics, Lomonosov Moscow State University, Moscow, Russia

^13^ Laboratory of Biological Structure Mechanics (LaBS), Department of Chemistry, Materials and Chemical Engineering “Giulio Natta”, Politecnico di Milano, Milano, Italy

^14^ College of Engineering and Informatics, National University of Ireland Galway, Ireland; National Centre for Biomedical Engineering Science, National University of Ireland Galway, Galway, Ireland

^15^ Cerenovus, Galway Neuro Technology Centre, Galway, Ireland

^16^ Institute of Biomedical Engineering, Department of Engineering Science, University of Oxford, Oxford, United Kingdom
